# Characterization of differential patient profiles and therapeutic responses of pharmacy customers for four ambroxol formulations

**DOI:** 10.1186/s40360-018-0229-y

**Published:** 2018-07-04

**Authors:** Peter Kardos, Kai-Michael Beeh, Ulrike Sent, Tobias Mueck, Heidemarie Gräter, Martin C. Michel

**Affiliations:** 1 Group Practice, Center for Allergy, Respiratory and Sleep Medicine, Red Cross Maingau Hospital, Frankfurt am Main, Germany; 2 insaf Respiratory Resarch Institute, Wiesbaden, Germany; 3 Medical Affairs Consumer Healthcare, Sanofi-Aventis Deutschland GmbH, Frankfurt-Hoechst, Germany; 4 Department of Pharmacology, Johannes Gutenberg University, Obere Zahlbacher Str. 67, 55131 Mainz, Germany

**Keywords:** Ambroxol, Acute cough, Pharmacy setting, Non-interventional study

## Abstract

**Background:**

Ambroxol relieves cough symptoms based on its secretagogue, anti-inflammatory, anti-oxidant, anti-bacterial, anti-viral, immunomodulatory and local anesthetic effects. The present study was designed to explore differential patient profiles and efficacy against acute respiratory symptoms of four formulations registered as over-the-counter medicines.

**Methods:**

Nine hundred sixty-five pharmacy customers purchasing one of four branded ambroxol formulations (extended release capsules, adult syrup, pediatric syrup and soft pastilles) filled a questionnaire including a patient-adapted version of the Bronchitis Severity Scale, several questions on degree of impairment by acute cough, time to onset of symptom relief and duration of treatment. Data on pediatric syrup users were entered by their parents. Based on the exploratory character of the study, no hypothesis-testing statistical analysis was applied.

**Results:**

Users of the pediatric syrup and the pastilles reported somewhat less severe baseline symptoms. The patient-adapted Bronchitis Severity Scale proved feasible as a self-administered tool. Among BSS items, ambroxol formulations improved chest pain while coughing to the largest and sputum to smallest degree (− 75% vs. -40%). Reported efficacy was comparable among formulations with minor differences in favor of the pediatric syrup. Time to onset of symptom relief was less than 60 min in more than 90% of patients and occurred prior to known systemic t_max_. Time to onset was the parameter with the greatest differences between formulations, being reported fastest with pastilles and pediatric syrup and, as expected, slowest with extended release capsules. All ambroxol formulations were well tolerated.

**Conclusions:**

We conclude that over-the-counter formulations of ambroxol exhibit comparable user profiles and efficacy. Differences in speed of onset of symptom relief may involve not only those in systemic pharmacokinetics but also local anesthetic effects of immediate release formulations. Differences between pediatric and adult syrup may in part reflect reporting bias.

## Background

The secretolytic agent ambroxol, a metabolite of bromhexine, enhances mucus clearance, facilitates expectoration and eases productive cough based on secretagogue activity, stimulation of pulmonary surfactant production and stimulation of mucociliary transport [1, 2]. It also has anti-inflammatory and anti-oxidant [3, 4] and anti-bacterial and anti-viral properties [2, 5]. Recently, ambroxol was shown to also exhibit immunomodulatory effects in a murine asthma model, where it normalized airway hyperresponsiveness and reduced eosinophils and Th2-related cytokines in bronchoalveolar lavage [6]. Finally, ambroxol affects lysosomal function, which may be beneficial in lysosomal storage diseases [7] or Parkinson’s disease [8], but the relevance of this finding remains to be tested clinically. While all of the above effects are assumed to occur by a systemic action, ambroxol also has local anesthetic effects, which are mediated by blockade of Na^+^ channels in the cell membrane [9, 10] and probably responsible for its effects in the treatment of sore throat.

Ambroxol is registered for secretolytic treatment of acute and chronic bronchopulmonary diseases associated with a disturbance of mucus formation and transport in adults and children. While many of the clinical studies demonstrating the efficacy and tolerability of ambroxol have been generated prior to the introduction of Good Clinical Practice, a recent review has identified 92 clinical studies of acceptable quality [4]. Randomized, placebo-controlled short-term (up to 2 weeks of treatment) studies showed efficacy against endpoints such as ease of expectoration, phlegm loosening, sputum volume and sputum viscosity [11–13]. Moreover, more recent studies have demonstrated the efficacy of ambroxol lozenges in the treatment of sore throat [14].

Since originally obtaining marketing authorization in Germany in 1978, ambroxol became available in many countries and in multiple formulations. Some of them have meanwhile become available as over-the-counter medications. These include branded formulations of extended release (ER) capsules, pastilles, and syrups for adult and pediatric use. The pediatric and adult syrup formulations are identical in composition except for aromas. This multitude of formulations in part reflects medical science, i.e. that syrups inherently are more effective against cough than tablets – irrespective of their active pharmacological ingredients [15]; it also reflects customer preferences and commercial considerations.

## Methods

The present study was designed to explore the different profiles of patients obtaining various ambroxol-containing formulations (Mucosolvan®; ER capsules, soft pastilles, adult syrup or pediatric syrup) as over-the-counter medications and their respective efficacy and tolerability against acute respiratory symptoms within the given indication. In line with the exploratory character of the survey, there was no a-priori hypothesis which formulation may be more effective for which type of patient. Additional goals were to explore a patient-adapted version of the Bronchitis Severity Scale (BSS) [16, 17] for use in a pharmacy setting, efficacy relative to duration of treatment, and treatment satisfaction. Finally, we aimed to obtain additional information on the tolerability of the four formulations of ambroxol based on real world evidence.

In this observational study, a total of 126 participating pharmacies were asked to invite customers aged 18 years or older having purchased one of the four products containing branded formulations of ambroxol (ER capsules, soft pastilles, adult syrup or pediatric syrup) to participate in an anonymous survey. Participants purchased the respective product on their own or according to the pharmacist’ recommendation. In case of a parent purchasing the pediatric syrup for a child, the parent was asked to participate on behalf of the child. The study protocol had instructed pharmacies only to invited customers to participate in the survey after a purchase decision had been made. Each pharmacy could recruit up to three customers per formulation. Recruitment was between 7.10.2016 and 4.5.2017. Participants received a € 5 coupon for future purchases from an online retailer as compensation for time spent filling the survey.

Precondition for participation was the purchase of one of the four ambroxol-containing products intended for current treatment of own common respiratory symptoms within the given indication or, for pediatric syrup, those of a child; the person purchasing the product had to have an age ≥ 18 years and be willing and able to independently, plausible and timely complete the questionnaire. The participants could return the survey in a sealed envelope either to the pharmacy or send it postage-free to a contract research organization. The package leaflet and/or the consulting pharmacist provided instructions on appropriate use.

The survey captured demographics and baseline symptoms of the participants prior to start of treatment. Captured demographic variables included gender, age and smoking status (smoking status of parent for pediatric syrup users). Symptom severity was assessed by a patient-adapted version of the BSS, a validated score which rates five key symptoms (cough, sputum, rattles (replacing rales on auscultation), chest pain while coughing and dyspnea) on a scale from 0 to 4 [16, 17]. Additional questions asked for the lead symptom (choice of dry cough, cough with moderate sputum (< 1 teaspoon/day), cough with much sputum (≥ 1 teaspoon/day) and cough bouts), daytime cough frequency (0–2, 3–4 and > 4 times/h), nightly awakening due to cough (0, 1, 2, 3 or ≥ 4 times/night), and degree of impairment for four conditions (falling asleep, exhaustion, ability to concentrate, performing daily tasks; each rated on a Likert scale from 0 to 3 as applies fully, applies mostly, applies partly or does not apply).

The survey also captured when treatment was started relative to onset of symptoms (upon first signs of cough, on day 1–2, on day 3–5 or on day 6), how long it was used (1, 2, 3, 4 or 5 days) and how quickly improvement of symptoms was noticed (0–15, > 15–30, > 30–60 and > 60 min or very fast, fast, moderately fast, slow). It also asked for BSS, frequency of cough, frequency of waking up due to cough in the night, and degree of impairment for five conditions (see above). Final questions were related patient assessment of global treatment success, tolerability and satisfaction with treatment.

Information on adverse events (AE) was collected based on a very broad operational definition as pre-specified in the study protocol. This included reporting of any AE via a healthcare professional on the AE form provided to the participating pharmacies; worsening of any item in the disease symptom or disease-associated impairment score (except for cough with expectoration, which is part of the mechanism of action of ambroxol); global efficacy or global tolerability rated as “poor”; deviation from package leaflet with regard to age range (< 12 years for ER capsules, < 6 years for pastilles).

Data analysis was performed using SAS (version 9.2, SAS Institute Inc., Cary, NC, USA). If one or two of the five BSS items had not been provided, the total BSS was extrapolated from the available items; other missing data were not replaced. In line with the exploratory character of the analyses and recent recommendations [18], no hypothesis-testing statistical analyses were performed. Data on categorical variables are reported as % of participants exhibiting a given parameter. Data on quantitative parameters are reported as means ± SD. Data collection and analysis was performed by Winicker Norimed GmbH (Nuremberg, Germany), a contract research organization, based on a statistical analysis plan developed by the authors. Ethical committee approval was neither required nor recommended for this type of research in Germany at the time it was performed.

## Results

### Baseline data

**Fig. 1:**
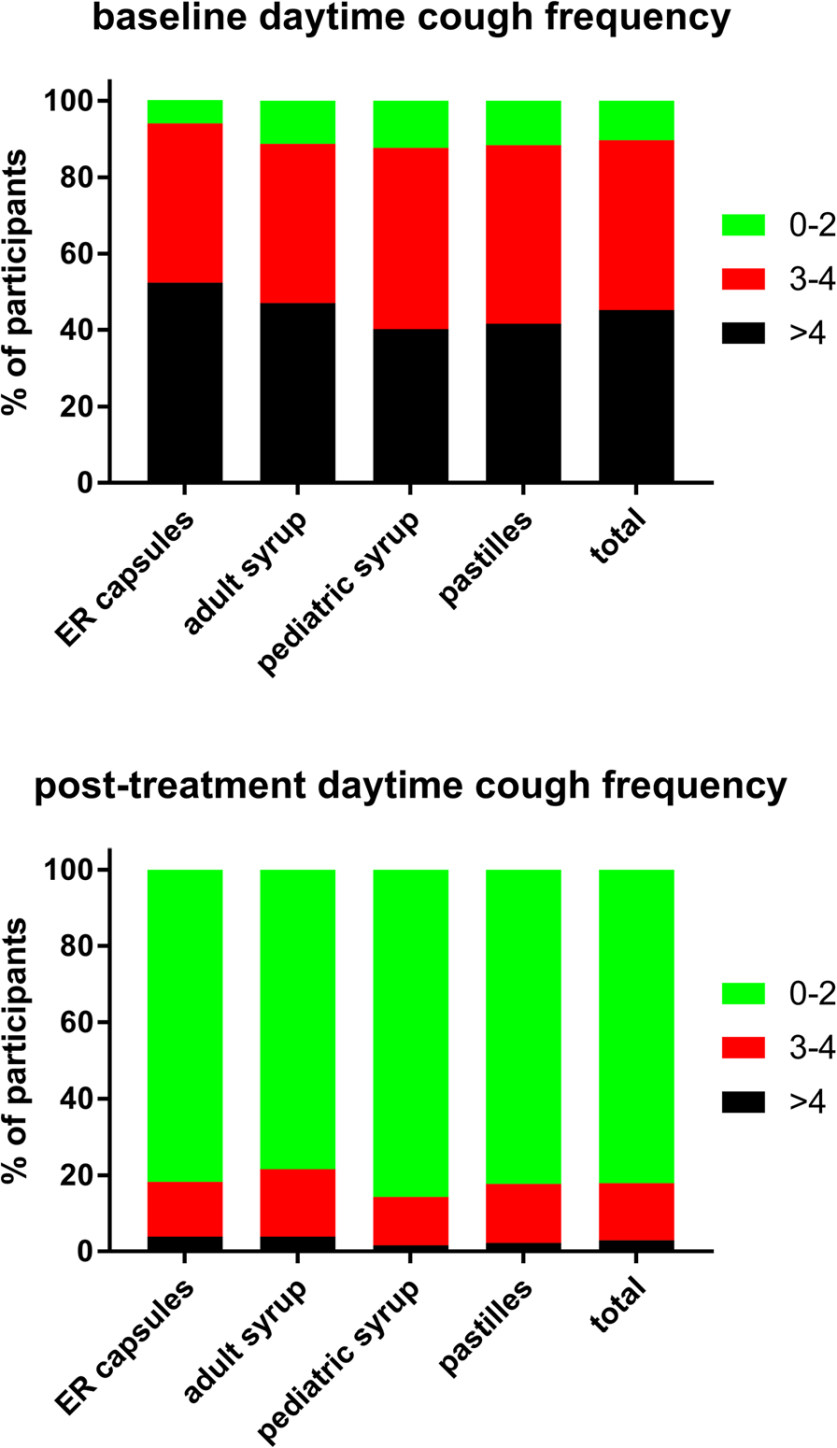
Daytime coughing frequency (coughs/h) before (upper panel) and after treatment (lower panel) with individual ambroxol preparations and in the overall group. Data are % of participants within a category

**Fig. 2:**
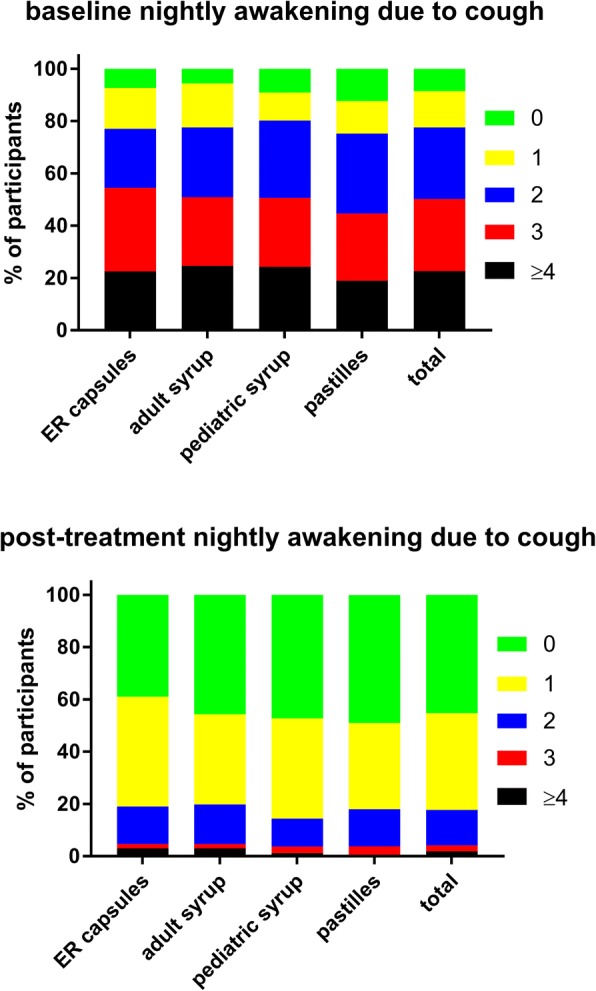
Nightly awakening due to cough (awakenings/night) before (upper panel) and after treatment (lower panel) with individual ambroxol preparations and in the overall group. Data are % of participants within a category

**Fig. 3:**
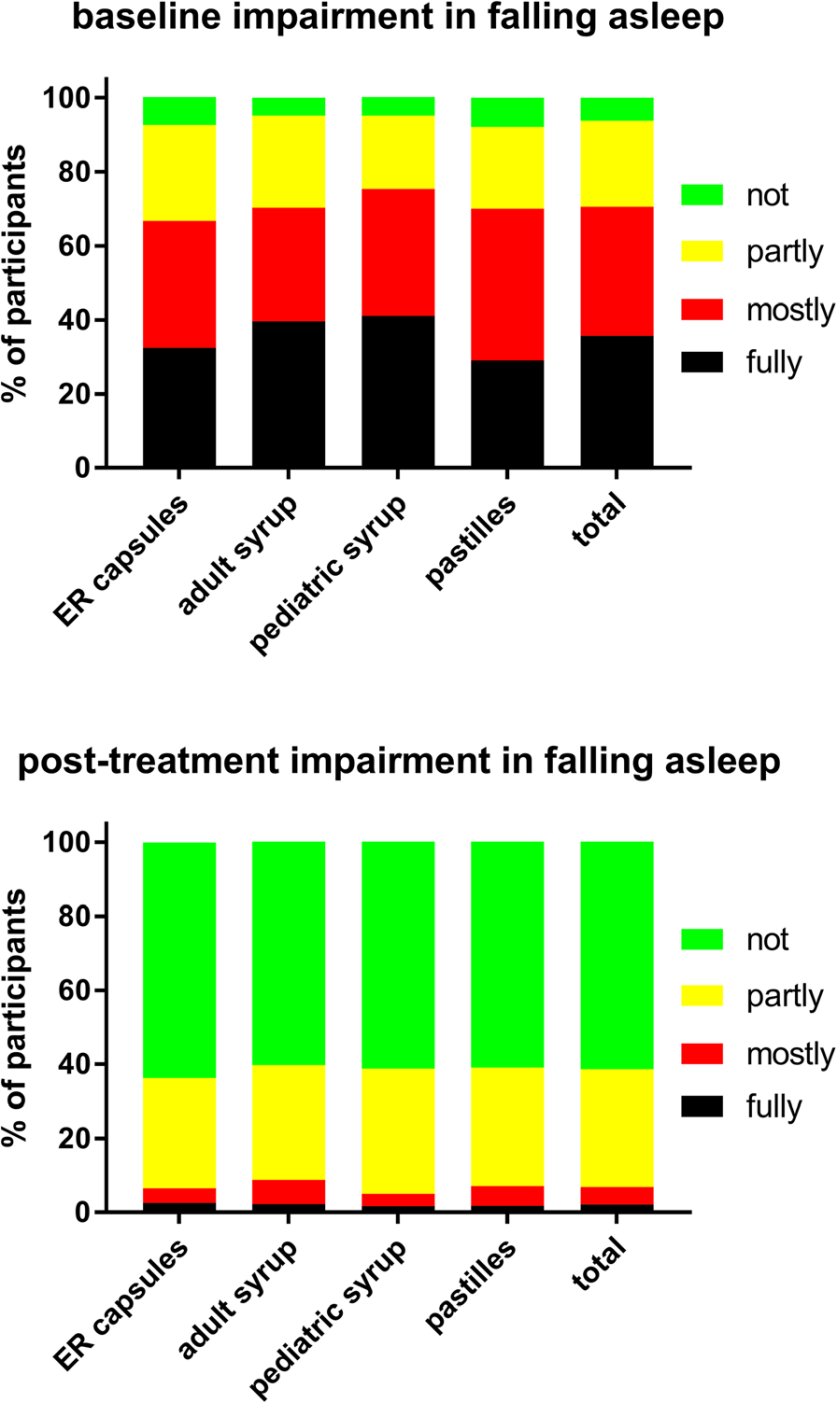
Impairment of ability to fall asleep due to cough (upper panel) and after treatment (lower panel) with individual ambroxol preparations and in the overall group. Data are % of participants within a category

**Fig. 4:**
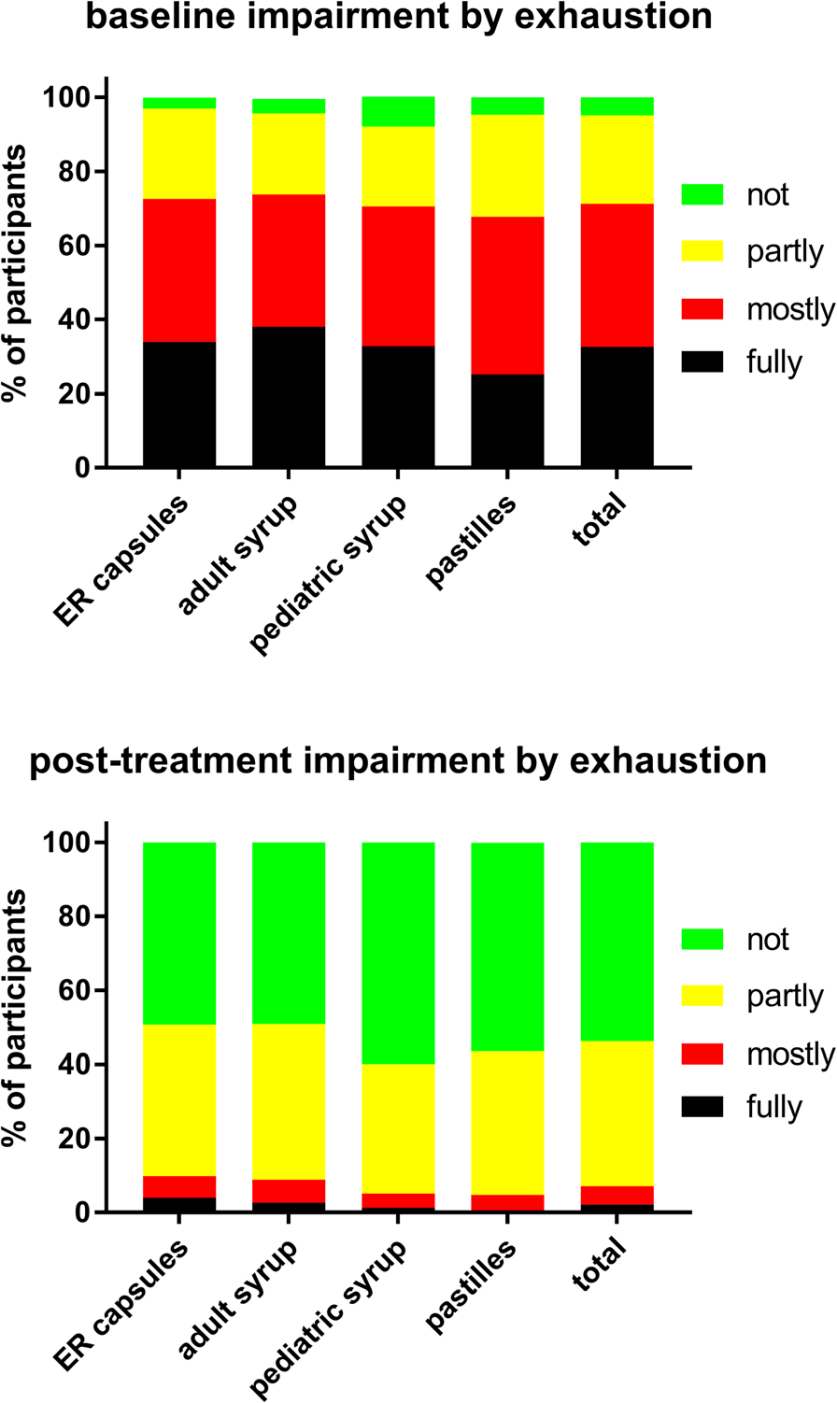
Impairment by exhaustion before (upper panel) and after treatment (lower panel) with individual ambroxol preparations and in the overall group. Data are % of participants within a category

**Fig. 5:**
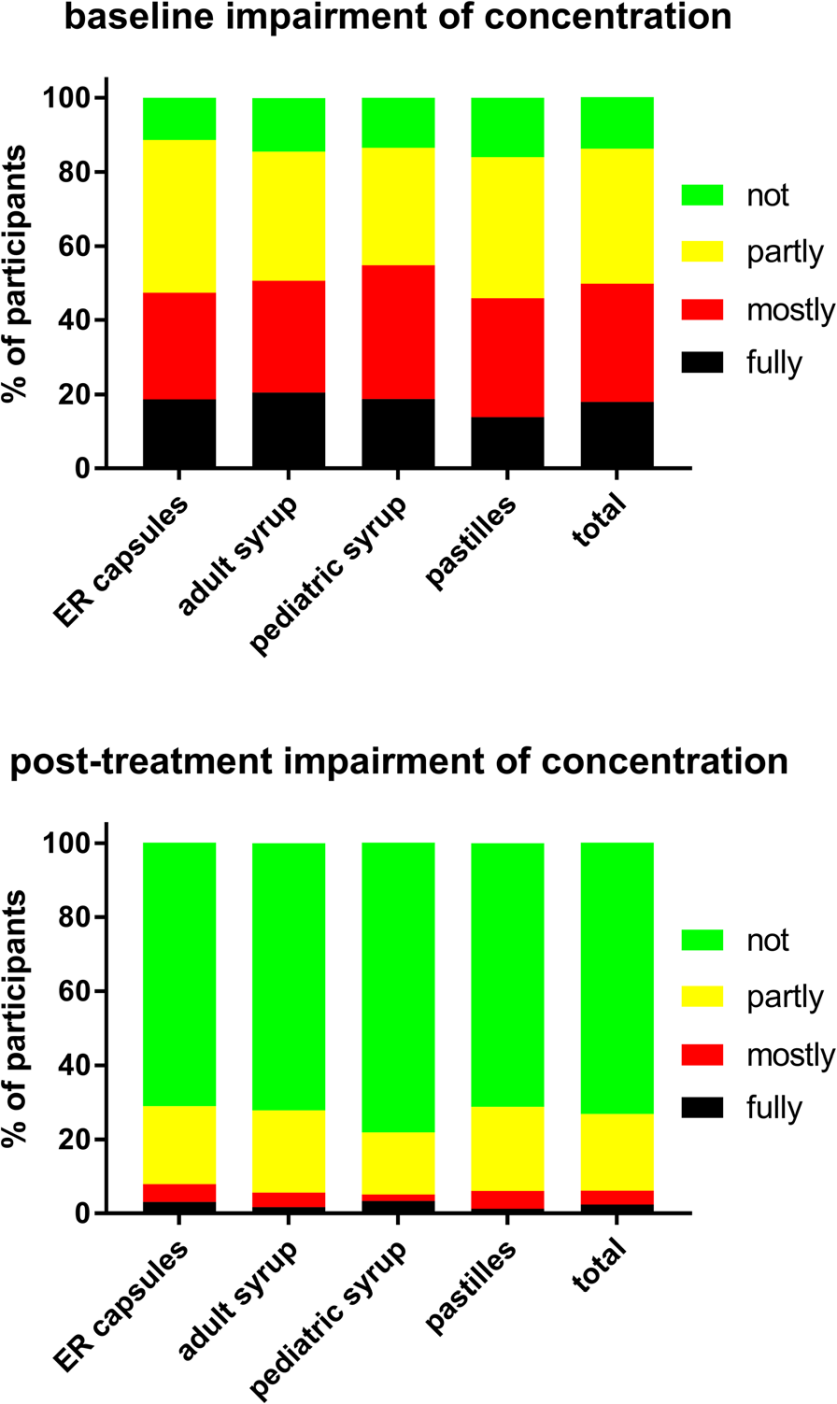
Impairment of ability to concentrate before (upper panel) and after treatment (lower panel) with individual ambroxol preparations and in the overall group. Data are % of participants within a category

**Fig. 6:**
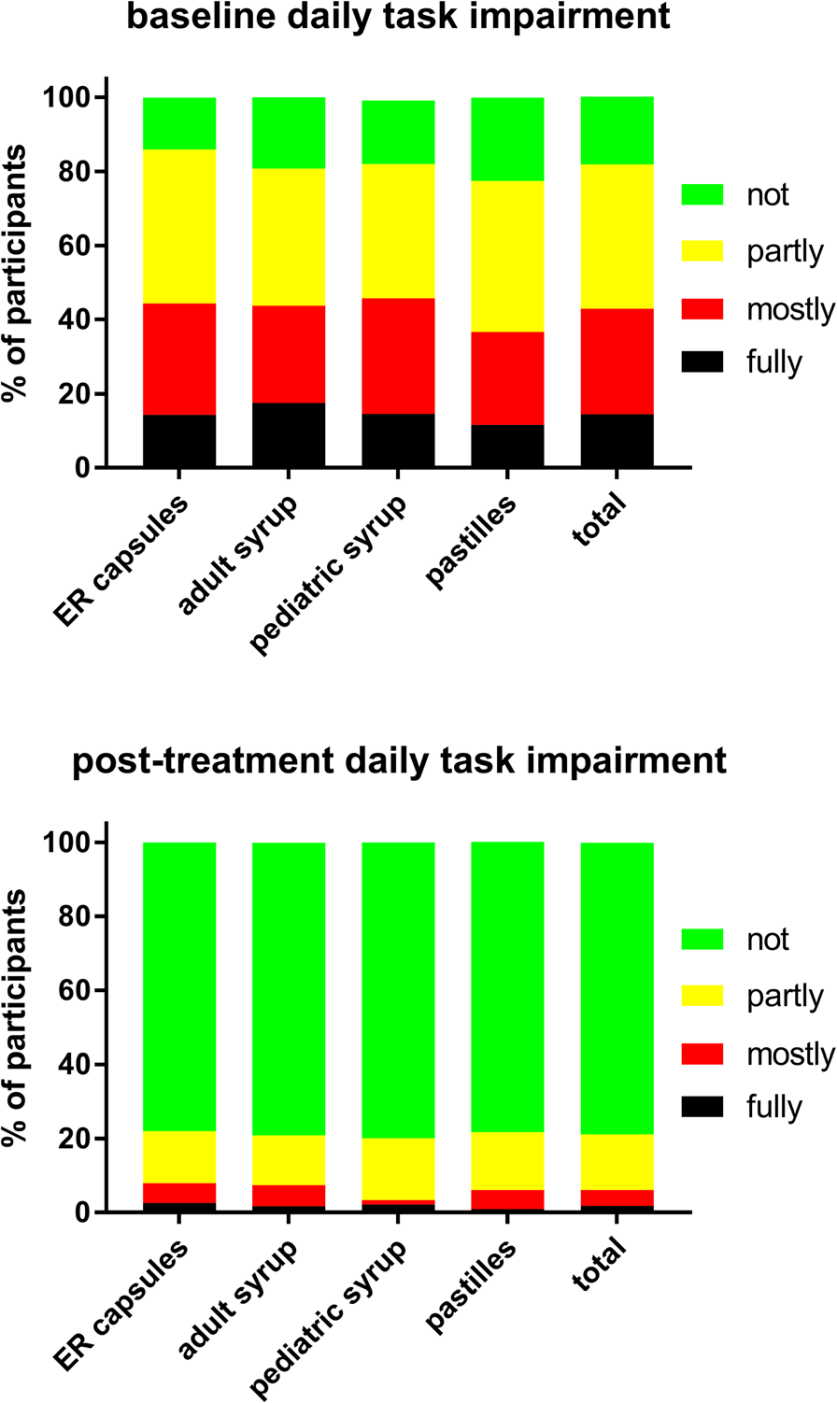
Impairment in ability to execute daily tasks before (upper panel) and after treatment (lower panel) with individual ambroxol preparations and in the overall group. Data are % of participants within a category

A total of 965 customers participated in the survey, equally distributed across the four formulations (Table 1). Twenty-four users were excluded from all analyses due to strong suspicion of incorrect answers; therefore, all analyses are based on a total of 941 subjects. Key demographic data are shown in Table 1. Users of the four ambroxol preparations differed somewhat in baseline symptom severity as determined by the adapted BSS (Table 2). Thus, users of the ER capsules and the adult syrup had a similar total BSS (10.0 and 10.1), whereas users of the pediatric syrup and pastilles were similar to each other but had a lower total BSS (8.7 and 8.8). While all four groups reported similar cough and sputum, the users of the ER capsules and adult syrup had more chest pain while coughing and more dyspnea. In the total cohort, total BSS increased with age (0–5 years: 8.2 ± 3.6, 6–11 years: 8.1 ± 3.1, 12–17 years: 9.3 ± 3.7, ≥18 years: 10.2 ± 3.8), which was largely driven by an age-dependent worsening of expectorations and chest pain while coughing. In the group of all ambroxol users, the most frequent lead symptom was cough with moderate sputum reported by 40.5%, cough with much sputum by 30.1%, cough bouts by 16.4% and dry cough by 13.0%. Daytime coughing frequency and number of nightly awakenings due to cough were comparable across ambroxol formulations; if anything, ER capsule users reported a somewhat greater frequency of both (Figs 1 and 2). Participants reported the strongest impairment in the ability to fall asleep due to cough (Fig. 3) and for exhaustion (Fig. 4), followed by ability to concentrate (Fig. 5) and least impairment for ability to execute daily tasks (Fig. 6). Users of the four ambroxol preparations reported comparable degrees of impairment; if anything, use of the pediatric syrup was associated with the largest impairment of ability to concentrate and to fall asleep.

**Table 1:** Demographic data of participating subjects. Note that smoking status for the pediatric formulation refers to that of parent (most heavily smoking one if different between parents). Data are means ± SD or percentages of given group

	ER capsules	Adult syrup	Pediatric syrup	Pastilles	Total
n	231	233	244	233	941
Gender, % male	38.0	31.8	40.9	38.2	37.2
Age, years	41.2 ± 15.4	39.3 ± 17.5	12.8 ± 12.8	35.7 ± 15.1	32.0 ± 19.1
Smoking status, % regular/occasional/non-smoker	22.4/17.5/60.1	21.6/18.9/59.5	4.5/8.0/87.5	17.2/12.7/70.1	18.1/15.2/66.7

**Table 2:** Baseline data, end of treatment data and intra-individual change of items and total score of the Bronchitis Severity Scale (BSS). Possible maximum for individual items is 4, for total score 20. Data are mean ± SD. Numbers of responders for a given item differed somewhat between items but ranged between 92 and 99% in all cases

	ER capsules	Adult syrup	Pediatric syrup	Pastilles	Total
Baseline data					
Cough	2.9 ± 0.7	2.9 ± 0.8	2.9 ± 0.7	2.7 ± 0.7	2.8 ± 0.7
Sputum	2.2 ± 1.0	2.1 ± 1.0	1.9 ± 1.0	2.0 ± 1.0	2.0 ± 1.0
Rattles	1.8 ± 1.1	1.9 ± 1.1	1.6 ± 1.1	1.4 ± 1.1	1.7 ± 1.1
Chest pain while coughing	1.8 ± 1.1	1.8 ± 1.1	1.4 ± 1.1	1.6 ± 1.1	1.6 ± 1.1
Dyspnea	1.5 ± 1.1	1.5 ± 1.2	1.0 ± 1.0	1.1 ± 1.1	1.3 ± 1.1
Total score	10.0 ± 3.8	10.1 ± 3.9	8.7 ± 3.5	8.8 ± 3.8	9.4 ± 3.8
Post-treatment data					
Cough	1.4 ± 0.7	1.3 ± 0.7	1.2 ± 0.6	1.2 ± 0.7	1.3 ± 0.7
Sputum	1.4 ± 1.0	1.3 ± 1.0	1.1 ± 0.9	1.1 ± 1.0	1.2 ± 1.0
Rattles	0.6 ± 0.8	0.6 ± 0.8	0.4 ± 0.7	0.5 ± 0.7	0.5 ± 0.7
Chest pain while coughing	0.6 ± 0.8	0.6 ± 0.8	0.3 ± 0.6	0.5 ± 0.7	0.5 ± 0.7
Dyspnea	0.5 ± 0.7	0.5 ± 0.7	0.3 ± 0.6	0.4 ± 0.6	0.4 ± 0.7
Total score	4.5 ± 3.0	4.1 ± 3.1	3.3 ± 2.5	3.7 ± 2.9	3.9 ± 2.9
Intra-individual change					
Cough	−1.5 ± 0.9	−1.6 ± 0.9	−1.7 ± 0.8	−1.5 ± 0.8	−1.6 ± 0.9
Sputum	−0.8 ± 1.4	−0.9 ± 1.4	−0.8 ± 1.3	−0.8 ± 1.3	−0.8 ± 1.3
Rattles	−1.2 ± 1.0	−1.3 ± 1.0	− 1.2 ± 1.0	− 0.9 ± 0.9	−1.2 ± 1.0
Chest pain while coughing	−1.2 ± 1.1	−1.3 ± 1.0	− 1.0 ± 1.0	−1.1 ± 0.9	− 1.2 ± 1.0
Dyspnea	−1.0 ± 1.0	− 1.0 ± 0.9	− 0.7 ± 0.9	− 0.8 ± 0.9	− 0.9 ± 0.9
Total score	−5.5 ± 3.8	−6.0 ± 3.8	−5.4 ± 3.3	−5.2 ± 3.3	−5.5 ± 3.6

### Treatment outcomes

Across all ambroxol formulations, treatment started upon first signs of cough in 9.1% of patients, on day 1–2 in 51.1%, on day 3–5 in 33.6% and on day 6 or later in 6.2%, indicating its primary use for the treatment of acute cough in line with the intention of the study. Start of treatment was comparable among preparations, but tended to be earlier with pediatric syrup and pastilles than with adult syrup or ER capsules (day 1–2: 59.0 and 50.6% vs. 46.8 and 47.6%, day 3–4: 31.1 and 21.2% vs. 36.9 and 37.2%). Mean duration of treatment was 4.3 ± 0.9 days, with little difference between formulations, also supporting the idea that these over-the-counter formulations of ambroxol are largely used for treatment of acute cough.

Time to start of symptom relief among all participants was within 1–15 min in 12.2% of patients, within 15–30 min in 38.4%, 30–60 min in 37.2% and > 60 min in 12.1%. While most users of the pediatric syrup (43.4%) and of the pastilles (45.2%) reported a time to onset of 15–30 min, most users of the adult syrup (42.1%) and the ER capsules (47.8%) reported start of symptom relief within 30–60 min. Correspondingly, a subjective start of symptom relief was reported as very fast, fast, moderately fast and slow in 11.1, 47.6, 34.4 and 6.9%, respectively. In comparison of the preparations, a very fast start of improvement was reported most frequently with the pediatric syrup (11.6%) and the pastilles (19.6%) and less frequently with adult syrup (7.8%) and the ER capsules (5.3%); correspondingly, a moderately fast start was reported most frequently with the adult syrup (35.3%) and the ER capsules (43.0%) and less frequently with the pediatric syrup (31.8%) and the pastilles (27.8%).

Across all formulations, treatment with ambroxol reduced the BSS by 5.5 points (mean end-of-treatment score), i.e. by 59% (Table 2). The strongest improvements were reported for chest pain while coughing (− 1.2; 75%), followed by rattles (− 1.2; 71%), dyspnea (− 0.9; 69%) and cough (− 1.6; 57%), whereas sputum was reduced least (− 0.8; 40%). Compared to ER capsules and pastilles (59%), the improvement was slightly larger with pediatric syrup (62%) and slightly smaller with adult syrup (55%). A similar pattern was observed for each item of the BSS. A post-hoc analysis compared the number of responders as defined by an at least 20, 30% or 40% reduction of the BSS between ambroxol formulations. Responder rate for the 20% reduction was 88.6, 89.5, 92.1 and 91.6% for ER capsules, adulty syrup, pediatric syrup and pastilles, respectively. Corresponding numbers for a 30% reduction were 80.3, 82.0, 87.1 and 84.5%, and for a 40% reduction 70.7, 73.7, 80.9 and 73.0%.

The frequency of daytime coughing was markedly reduced by all ambroxol formulations (Fig. 1). Among all participants, 82.1% reported 0–2 coughs/h, 15.0% 3–4 coughs/h and 2.9% > 4 coughs/h after treatment. Notably, 21.2% of subjects with > 4 coughs/h at baseline went to 3–4 and 74.3% to 0–2 coughs/h; similarly, 87.5% of those with 3–4 coughs/h at baseline went to 0–2. The number of nightly awakenings due to cough was also markedly reduced by all ambroxol formulations (Fig. 2). Notably, 31.6% of patients with ≥4 awakenings/night at baseline went to 2, 33.0% to 1 and 23.1% to 0 awakenings/night; similarly, 15.8% of those with 3 awakenings/night went to 2, 56.4% to 1 and 23.9% to 0 awakenings/night. This shift pattern for both daytime coughing frequency and nightly awakening was comparable for all ambroxol preparations (Figs 1 and 2).

Treatment with all four ambroxol preparations markedly improved bronchitis-associated impairments, i.e. ability to fall asleep due to cough (Fig. 3) and exhaustion (Fig. 4), followed by ability to concentrate (Fig. 5) and least impairment for ability to execute daily tasks (Fig. 6). Thus, 51.4% of those reporting to have a fully impaired ability to fall asleep due to cough at baseline across all ambroxol formulations had no impairment at all after treatment (Fig. 3). Similarly, 45.1% reporting being fully impaired by exhaustion had no impairment after treatment (Fig. 4), 55.1% reporting being fully impaired in their ability to concentrate had no impairment after treatment (Fig. 5) and 60% reporting fully impaired in their ability to perform daily tasks had no impairment after treatment (Fig. 6).

**Fig. 7:**
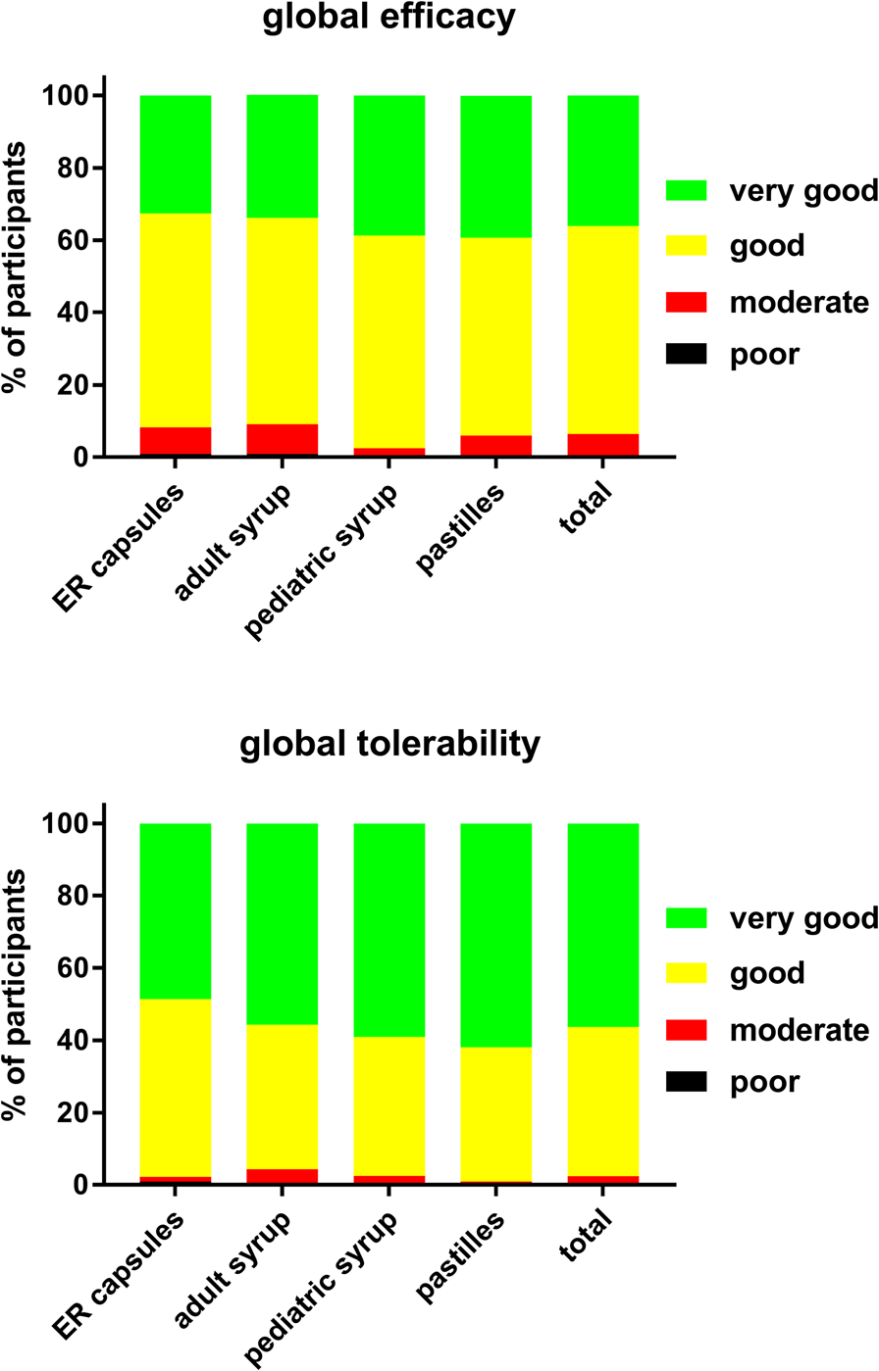
Patient-reported global efficacy and tolerability with individual ambroxol preparations and in the overall group. Data are % of participants within a category

Participants rated global efficacy of ambroxol across formulations as very good, good, moderate or poor in 36.1, 57.5, 6.0 and 0.4% of cases, respectively (Fig. 7). While these estimates were comparable across formulations, ratings were slightly more favorable for the pediatric syrup and the pastilles (Fig. 7). Interestingly, the percentage of patients rating efficacy as very good increased with duration of treatment; thus, it was 31.0% in 2-day users, 34.0% in 3-day users, 34.4% in 4-day users and 38.0% in 5-day users. This trend was similarly observed with all formulations. Patients rated global tolerability of ambroxol as very good, good, moderate or poor in 56.4, 41.2, 2.1 and 0.3% of cases, respectively (Fig. 7). While these estimates were comparable across preparations, ratings were slightly more favorable for the pastilles and the pediatric syrup (Fig. 7).

Based on the very broad operational definition of AE (see Methods), AEs were registered in 99 patients (10.3%); none of them had a fatal outcome or was considered serious. This included two cases of diarrhea (0.2%), three with poor global tolerability (0.3%) and four with poor global efficacy (0.4%); the other 90 cases were based on worsening of items in the symptom and impairment scores.

## Discussion

Ambroxol has multiple mechanisms of action; while most of them including secretolytic, anti-inflammatory and anti-oxidant activity are assumed to occur by systemic exposure [4], Na^+^ channel blockade in the treatment of sore throat is assumed to occur by a local effect [9, 10]. However, symptom reduction by syrup and pastilles may partly also involve local effects, for instance in the pharynx as part of the cough inhibition. Against this background, we have surveyed pharmacy customers obtaining different formulations of ambroxol as over-the-counter medication to explore specific profiles of patient groups selecting these formulations as well as their corresponding efficacy and tolerability in a real-world setting.

### Critique of methods and feasibility

Our data are based on a survey of pharmacy customers purchasing one of four branded ambroxol containing products. The resulting data are not expected to have the same quality as those collected by a physician or other healthcare professional. Moreover, the use of anonymous reponses implies that source data verification was not possible. However, we deem this setting suitable to obtain real-world evidence for over-the-counter medications. Moreover, our previous work using other airway-related over-the-counter medications has demonstrated the external validity of this approach for generating real-world evidence for non-prescription medicines [19, 20].

Our study design did not include a control group, e.g. placebo, for three reasons: Firstly, the efficacy and tolerability of ambroxol has been demonstrated in numerous controlled trials [4]. Therefore, there was no need to re-establish this using a placebo group. Second, such a control group would have interfered with the non-interventional character of our study. Third, the primary intention of our survey was the comparison between ambroxol formulations. Therefore, our data should not be interpreted as proof of efficacy or tolerability but rather as complementary to previously reported controlled studies. It flows from the non-interventional character of our study that we do not have specific data on ingested doses. While we can assume that ambroxol administration was in line with dosing recommendations of the package insert, this limits comparison of formulations based on exposure data. As the primary aim of the study was explorative, no a priori hypotheses existed; accordingly, no hypothesis-testing statistical tests were applied based on recent recommendations [18].

We have used a patient-adapted version of the BSS to obtain key data (Table 2). The BSS has been validated as a tool for controlled trials [16, 17]. One of the items in the BSS is “rales on auscultation”, which obviously needs to be assessed by a healthcare professional. As this would counter the intention to generate real-world evidence, we have replaced this item by the subjective patient-assessed symptom of “rattles”. Our data show that pharmacy customers can use this adapted version of the BSS as a self-administered tool.

Based on the efficacy and safety of ambroxol in controlled studies in both adults and children [4, 11–13], our study included a pediatric syrup which yielded somewhat different results than the adult syrup, particularly for time to onset of symptom relief. Several factors may have contributed to such reported differences. Firstly, the vast majority of the users were children and adolescents, but some users have been adults as indicated by the age of 12.8 ± 12.8 years of users of the pediatric syrup (Table 1). This does not represent inappropriate use because the pharmacologically active content is identical to the adult syrup and the package insert of the pediatric syrup also includes dosing recommendations for adults. Nonetheless, it limits interpretation of these data. Second, users of the pediatric syrup tended to have less severe baseline symptoms than those of the adult syrup. Third, the pathophysiology of cough may differ between children and adults. Forth, and perhaps most importantly, data on children were not reported by the patient but by their parents. Previous studies have shown that parental reporting of children’s cough may be biased, for instance smoking parents under-reported night-time cough in their children [21]. However, parent reporting of effects of cough on the quality of life in children has been validated as reliably according to other studies [22]. Therefore, interpretation of data obtained with the pediatric syrup needs to consider potential bias from inclusion of some adult users, differences in baseline severity as well as reporting bias.

### Baseline data

Among pooled users of all four ambroxol formulations, cough with moderate and with much sputum production were the most frequently mentioned lead symptoms, accounting for more than 70% of all patients, whereas only 13% reported dry cough as their lead symptom. Accordingly, patients rated the severity of cough higher than that of any other item of the BSS (Table 2). This is not surprising because 45% of participants reported to experience an average of four or more coughs per hour during daytime (Fig. 1). Similarly, 36 and 35% reported “full” or “mostly” impairment of falling asleep (Fig. 3). Awakening four or more times during the night due to coughing was reported in less than 25% of subjects (Fig. 2). While this indicates that coughing impairs falling asleep more frequently than staying asleep, it should be noted that patients experience nighttime cough as a much greater burden than daytime cough [23]. In contrast, users of ambroxol reported full impairment of their ability to concentrate or to execute daily tasks in only 18 and 15% of cases, respectively.

Among formulations for intended use in adults, there was a consistent trend that users of soft pastilles had the least severe symptoms at baseline, be it for the adapted BSS or any of the symptoms shown in Figs 1, 2, 3, 4, 5 and 6. This may reflect that pastilles generally are considered a rather mild form of treatment, perhaps even seen as acting purely locally, whereas capsules and adult syrup are perceived to act systemically. Such perception may have caused a selection bias among those with less severe symptoms.

### Treatment data

The present data as reported by pharmacy customers are in line with those from controlled clinical studies [4, 11–13], confirming the efficacy and tolerability of ambroxol in a real-world setting. Moreover, they demonstrate that the patient-adapted version of the BSS is suitable as a self-assessment tool. However, in the absence of reports on effects of ambroxol on BSS in controlled trials, this does not substitute for a formal validation of this version.

The primary aim of the present study had been a comparison of four ambroxol formulations. Focusing on the BSS as efficacy parameter and considering differences in baseline severity among user groups, efficacy was comparable between the formulations. If anything, the pediatric syrup was reported to be slightly more effective and the adult syrup slightly less effective. A similar pattern was also observed for the questions on impairment by the respiratory symptoms. Several factors may have contributed to minor differences in reported efficacy. These include local effects of syrups and pastilles related to local anesthetic action [9, 10], antitussive effects of syrups irrespective of active pharmacological ingredients [15, 24] as well as selection and reporting biases; factors specific for users of the pediatric syrup have been discussed above.

About 90% of participants reported the onset of effect to require no more than 60 min, showing a fast onset of all formulations. This is earlier than the systemic pharmacokinetic t_max_ of the ambroxol formulations, which is 1–2.5 h for all immediate release formulations [25] and 6.5 h for ER capsules [26]. This difference may in part be explained by local effects of the syrups and pastilles, but also points to a possible placebo component in reported time to onset of symptom relief.

Time to onset of symptom relief is the parameter differing most notably between formulations. Thus, compared to the adult syrup, users of pediatric syrup and pastilles reported a somewhat faster and those of the ER capsules a somewhat slower onset of symptom relief. A slower onset of action of the ER capsules is compatible with their later systemic pharmacokinetic t_max_ (6.5 vs. 1–2.5; [26]). A faster onset of action after ingestion of first dose with pastilles may be explained by a longer local contact time in the pharynx, allowing for a greater contribution of local anesthetic effects [9, 10]. Factors possibly involved in specific differential findings in users of the pediatric syrup have been discussed above.

Across all formulations, reported efficacy of treatment increased to some degree with duration of use, i.e. efficacy was rated as very good by 31.0% of subjects using ambroxol for 1 day only, with a step-wise increase to 38.0% in 5-day users. Based on a terminal elimination half-life of 7–11 h [25–27], pharmacokinetic steady-state is not expected to occur earlier than late on the second day of treatment, making earlier full efficacy unlikely. On the other hand, subjects stopping treatment after the first day (other than for adverse events, a situation not reported in this study) are likely to represent a biased sample of patients with very good symptom improvement. As symptoms of acute bronchitis may spontaneously resolve within few days, it is difficult to assess the relative roles of bias vs. reaching pharmacokinetic steady state in the absence of a placebo control group.

Tolerability assessment in a non-controlled study is difficult due to lack of a control group. This particularly applies if any worsening of symptoms or condition-associated impairments is counted as AE, as was done in our study. Such worsening accounted for most of the reported AEs. This is reflected by 97.3% of all participants rating global tolerability as very good or good and the overall improvements in symptom and associated impairment scores at the group level. Therefore, the tolerability data from the present study are not providing new signals or concerns relative to the established tolerability and safety of ambroxol.

## Conclusions

The present non-interventional, exploratory study based on effects reported by pharmacy customers confirms the efficacy and tolerability of ambroxol in controlled clinical studies [4, 11–13] in a real-world setting. It establishes that the patient-adapted version of the BSS is suitable for self-assessment, but its validity in this setting remains to be tested. Customers obtaining medicines with different formulations of ambroxol exhibit a differential qualitative and quantitative symptom profile, i.e. pastilles and the pediatric syrup being more often chosen by subjects with less severe symptoms. Nevertheless, efficacy and tolerability of the four formulations tested here was rather similar and, if anything, somewhat greater with pastilles and pediatric syrup, perhaps reflecting the lesser severity of baseline symptoms. We consider it likely that bias of patient perception is involved in these minor differences. The largest difference between formulations was reported time to onset of symptom relief; this may also at least partly reflect patient perceptions as it can only partly be explained by different pharmacokinetic profiles. In a more general view, our data support the concept that symptom relief in over-the-counter self-medication settings are likely to reflect a combination of pharmacodynamic effects and patient perception.

## Abbreviations

AE: Adverse event
BSS: Bronchitis severity scale
ER: Extended release
